# Intranasal delivery of mesenchymal stem cell secretome repairs the brain of Alzheimer’s mice

**DOI:** 10.1038/s41418-020-0592-2

**Published:** 2020-07-23

**Authors:** Giulia Santamaria, Edoardo Brandi, Pietro La Vitola, Federica Grandi, Giovanni Ferrara, Francesca Pischiutta, Gloria Vegliante, Elisa R. Zanier, Francesca Re, Antonio Uccelli, Gianluigi Forloni, Nicole Kerlero de Rosbo, Claudia Balducci

**Affiliations:** 1 Department of Neuroscience, Istituto di Ricerche Farmacologiche Mario Negri IRCCS, via Mario Negri 2, 20156 Milan, Italy; 2 DINOGMI, University of Genoa, Largo Daneo, 3, 16132 Genoa, Italy; 3 School of Medicine and Surgery, Nanomedicine Center NANOMIB, University of Milano-Bicocca, Via Raoul Follereau 3, 20854 Vedano al Lambro, MB Italy; 4 Ospedale Policlinico San Martino IRCCS, Largo Rosanna Benzi, 10, 16132 Genoa, Italy

**Keywords:** Neural ageing, Neurological disorders, Inflammation

## Abstract

The multiplicity of systems affected in Alzheimer’s disease (AD) brains calls for multi-target therapies. Although mesenchymal stem cells (MSC) are promising candidates, their clinical application is limited because of risks related to their direct implantation in the host. This could be overcome by exploiting their paracrine action. We herein demonstrate that *in vivo* systemic administration of secretome collected from MSC exposed in vitro to AD mouse brain homogenates (MSC-CS), fully replicates the cell-mediated neuroreparative effects in APP/PS1 AD mice. We found a complete but transient memory recovery by 7 days, which vanished by 14 days, after a single MSC-CS intravenous administration in 12-month or 22–24-month-old mice. Treatment significantly reduced plaque load, microglia activation, and expression of cytokines in astrocytes in younger, but not aged, mice at 7 days. To optimize efficacy, we established a sustained treatment protocol in aged mice through intranasal route. Once-weekly intranasal administration of MSC-CS induced persistent memory recovery, with dramatic reduction of plaques surrounded by a lower density of β-amyloid oligomers. Gliosis and the phagocytic marker CD68 were decreased. We found a higher neuronal density in cortex and hippocampus, associated with a reduction in hippocampal shrinkage and a longer lifespan indicating healthier conditions of MSC-CS-treated compared to vehicle-treated APP/PS1 mice. Our data prove that MSC-CS displays a great multi-level therapeutic potential, and lay the foundation for identifying the therapeutic secretome bioreactors leading to the development of an efficacious multi-reparative cocktail drug, towards abrogating the need for MSC implantation and risks related to their direct use.

## Introduction

Alzheimer’s disease (AD) is the main form of dementia in the elderly [1], urgently calling for a cure. AD brains show deposited extracellular senile plaques enriched in β-amyloid (Aβ) aggregates, and intracellular *tau*-enriched neurofibrillary tangles [2]. Neuropathology is complex and affects synaptic and cognitive function [3], as well as ion channels, mitochondrial function, and the vascular system [4]. AD brains are permanently inflamed, and released cytokines foster neurotoxicity culminating in gross brain atrophy [5, 6]. Although Aβ remains the main recognized culprit of AD, Aβ-centric therapies likely fail because the complex pathology needs multi-target approaches.

Mesenchymal stem cells (MSC) hold great promise as an alternative, multi-level approach in the therapy of intractable neurodegenerative disorders. MSC exert direct neuroprotective/neuroreparative effects through the release of neurotrophic factors and potent immunomodulatory properties [7]. Notably, MSC are the only stem cells endowed with anti-amyloidogenic activities [8]. MSC therapeutic potential has been documented in various AD animal models with data showing anti-inflammatory, anti-amyloid properties, and memory recovery [8, 9] and is being considered for several neurological diseases [10].

In spite of many advantages of MSC-based therapy, various challenges limit their clinical application, including: (i) invasive cell isolation process, (ii) loss of potency, (iii) limited lifespan, (iv) huge expansion costs [11], as well as the possibility, albeit low, of uncontrollable cell proliferation. It is well-accepted that infused MSC act through paracrine mechanisms by releasing bioactive components in their secretome when exposed to an injured environment, rather than through direct engraftment. In vitro and in vivo studies have shown both neuroprotective and anti-inflammatory effects exerted by MSC secretome itself in various disease models [11, 12], but not yet in AD mice.

We herein demonstrate that the secretome derived from MSC pre-conditioned in vitro in an AD environment (MSC-CS) fully replicates multiple neuroreparative activities exerted by implanted MSC themselves. Furthermore, we establish a preclinical, non-invasive and sustained treatment protocol leading to a reversal of functional and structural damage in aged AD mice. These findings hold enormous translational potential, since they provide the opportunity to circumvent the direct implantation of MSC and the limitations related to their clinical application.

## Results

### MSC-CS rapidly restores memory in 12-month-old APP/PS1 mice, albeit transiently

It is hypothesized that the crosstalk between MSC and the “injured” environment may be vital for the release of protective factors by MSC. Thus, we tested whether exposure of MSC to AD brain homogenate would have stimulated MSC to release a neuroreparative secretome. We, thus, compared the therapeutic efficacy of the secretome derived from MSC grown in standard conditions (MSC-UCS) with that derived from MSC exposed to brain homogenate from APP/PS1 mice (MSC-CS). The MSC-CS dose selected was that corresponding to 1 × 10^6^ MSC inducing memory recovery when IV injected in 11-month-old APP/PS1 mice (Fig. S1).

**Fig. 1:**
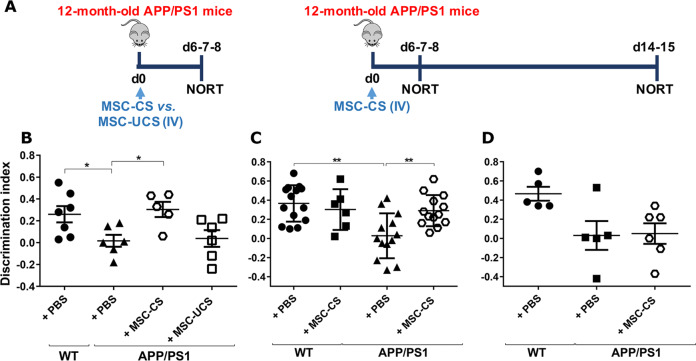
One IV injection of MSC-CS, but not MSC-UCS, transiently restores memory in 12-month-old APP/PS1 mice. **a** Experimental design. **b** Comparison of the discrimination index (DI) of APP/PS1 mice and their age-matched WT littermates receiving either PBS, MSC-CS or MSC-UCS and tested in the NORT. DI of WT and APP/PS1 mice treated with PBS or MSC-CS and tested in the NORT 7 days (**c**) and 14 days (**d**) post-injection. Data are expressed as scatter plots with mean ± SEM. One-way ANOVA in **b** and **d**. Two-way ANOVA in **c**, *P < 0.05; **P < 0.01 Tukey’s multiple comparison post-hoc test.

MSC-CS or MSC-UCS were intravenously (IV) injected in 12-month-old APP/PS1 mice tested 7 days post-injection in the novel object recognition memory test (NORT) (experimental design in Fig. 1a). A single MSC-CS IV administration induced a significant memory recovery in APP/PS1 mice. Of note, MSC-UCS administration was not effective (Fig. 1b). The efficacy of MSC-CS was fully confirmed in a second experiment, also showing that MSC-CS did not affect memory of WT mice (Fig. 1c).

When mice were retested in the NORT 14 days post-treatment, the overall effect of MSC-CS on memory was lost in 50% of MSC-CS-treated mice, thus indicating a transient efficacy of single-dose MSC-CS administration (Fig. 1d).

### A single MSC-CS IV injection is sufficient to decrease amyloidosis and neuroinflammation

**Fig. 2:**
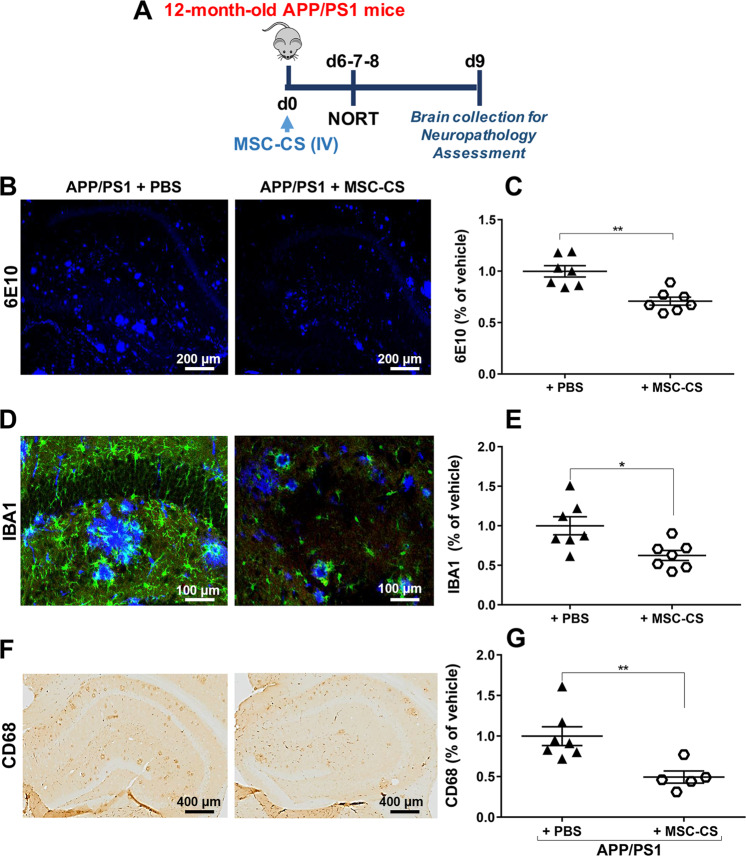
A single IV injection of MSC-CS reduces brain amyloidosis and microglial activation. **a** Experimental design. **b** Confocal images of the hippocampal area marked with the 6E10 anti-Aβ antibody, evidencing deposited amyloid plaques. **c** Plaque quantification. **d** Confocal images of the hippocampal area marked with both 6E10 for plaques and IBA1 for microglial cells. **e** IBA1-marked area quantification. **f** Hippocampal area immunostained for the phagocytic marker CD68 quantified in **g**. Data are expressed as scatter plots with mean ± SEM. *P < 0.05; **P < 0.01 Student’s *t* test.

Immunohistochemical analysis for amyloid plaques (Fig. 2b) on brain tissue from 12-month-old APP/PS1 mice treated with MSC-CS (experimental design in Fig. 2a) and plaque quantification (Fig. 2c) showed a 30% significant reduction in the number of total hippocampal and cortical Aβ plaques.

Aβ plaques in patients and mice are intimately surrounded by activated glial cells with a double sword action promoting plaque clearing [6, 13], but also neurotoxicity and memory impairment [14]. We thus evaluated the extent of hippocampal inflammation, a crucial brain area involved in recognition memory [15] and with prominent gliosis in APP/PS1 mice at this age. A co-localization of plaques and microglia well describing MSC-CS effect on the microglia pan marker IBA1 is shown in Fig. 2d. MSC-CS-treated APP/PS1 mice showed a significant reduction in IBA1 positivity (Fig. 2e), with residual cells displaying a resting state-like morphology. Treated APP/PS1 mice also showed a significant reduction in the CD68-marked area (Fig. 2f, g), a lysosomal glycoprotein associated with phagocytic function and lysosomal trafficking [16, 17], suggesting that spared plaques neither recall microglia nor exert a pro-inflammatory action.

**Fig. 3:**
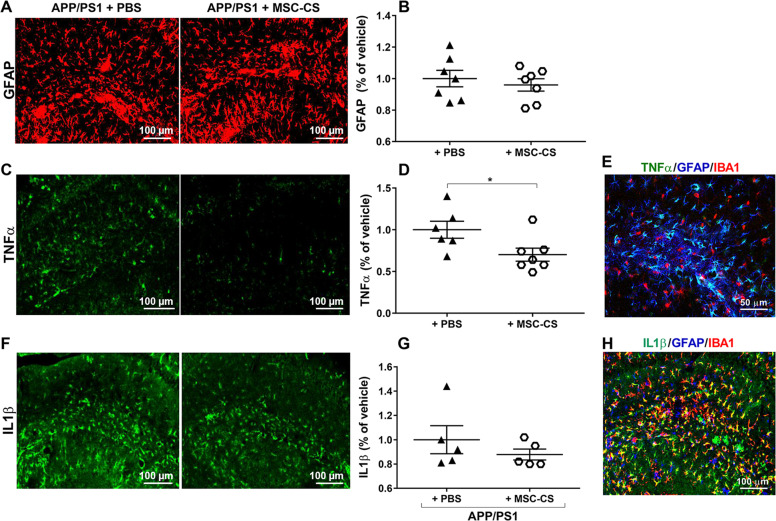
Single IV injection of MSC-CS improves the inflammatory phenotype of astrocytes. **a** Confocal images of hippocampal slices from MSC-CS-treated and PBS-treated mice immunostained for GFAP. **b** GFAP-marked area quantification evidencing the degree of astrogliosis between treatment groups. **c** Hippocampal slices immunostained for TNFα and relative quantification in **d**. **e** Merge of hippocampal immunostaining for TNFα-GFAP-IBA1. **f** Hippocampal immunostaining for IL1β quantified in **g**; **h** Merge of hippocampal immunostaining for IL1β-GFAP-IBA1. Quantification data are expressed as scatter plots with mean ± SEM. *P < 0.05, Student’s *t* test.

We then assessed the effect of MSC-CS on astrogliosis, another neuroinflammatory hallmark in AD patient [14] and mouse [5] brains, by measuring the expression of the anti-glial fibrillary acidic protein (GFAP) through histology. GFAP-marked area was not affected by the treatment (Fig. 3a, b). Notably, when the two most representative cytokines of AD, interleukin-1β (IL-1β) and tumor necrosis factor-α (TNFα) [18], were analyzed, we found a statistically significant 30% decrease in hippocampal TNFα expression (Fig. 3c, d) between MSC-CS- and PBS-treated APP/PS1, whereas the expression of IL-1β was unchanged (Fig. 3f, g). As both cytokines are mainly expressed by astrocytes in APP/PS1 mice (Fig. 3e and h), these data suggest that MSC-CS treatment partially reduced the pro-inflammatory astrocyte A1 phenotype [19].

### A single IV injection restores memory in 22-month-old APP/PS1 mice, but fails to improve neuropathology

In order to assess MSC-CS efficacy at a more severe stage of AD, we replicated our study in 22-month-old APP/PS1 mice. Again, a single IV injection of MSC-CS restored APP/PS1 recognition memory in the NORT 7 days post-treatment (Fig. S2A). Unexpectedly however, it did no affected amyloidosis or neuroinflammation (Fig. S2B, C), thus indicating that a single injection is sufficient to restore memory at this advanced age, but fails to modify other crucial biomarkers.

### Continuous, weekly, intranasal treatment with MSC-CS succeeds in imparting sustained memory recovery and repairing the neuropathology in 25-month-old APP/PS1 mouse brain

The transient functional recovery upon MSC-CS treatment and no improvement in neuropathology in aged mice suggested the need for a regular treatment to optimize the protocol. Due to limitations linked to repeated IV injections, we chose to exploit the intranasal (IN) route, which is non-invasive and offers the advantage of direct MSC-CS brain delivery via olfactory route [20].

**Fig. 4:**
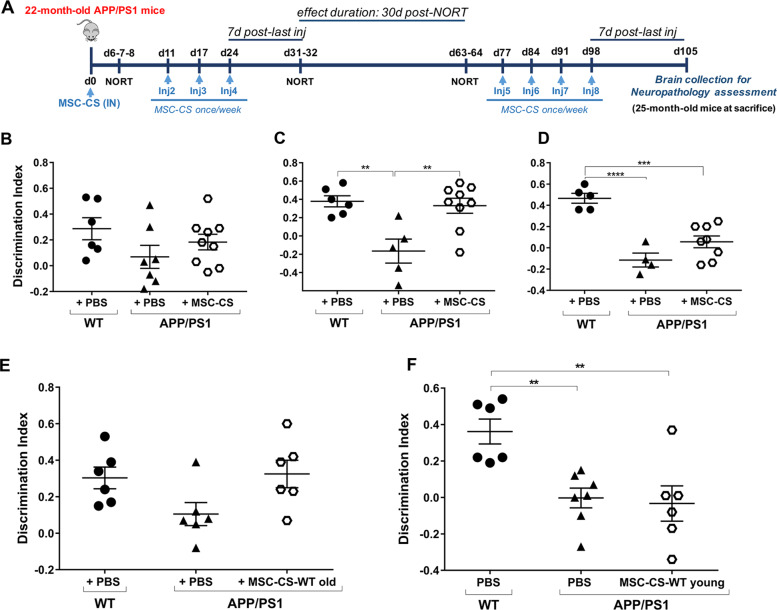
Repeated IN delivery of MSC-CS significantly restores memory whereas MSC-CS-WT old or young has partial or no effect, respectively. **a** Experimental design for MSC-CS treatment. DI of the recognition memory task of mice tested 7 days after 1 (**b**) or 4 (**c**) IN administrations, or 39 days after suspension of MSC-CS treatment (**d**). **e** DI of the recognition memory task of mice tested 7 days after 4 IN administrations of MSC-CS-WT old. **f** DI of the recognition memory task of mice tested 7 days after 4 IN administrations of MSC-CS-WT young. Data are expressed as scatter plots with mean ± SEM. One-way ANOVA; **P < 0.01; ***P < 0.001; ****P < 0.0001, Tukey’s multiple comparison post-hoc test.

Twenty-two-month-old APP/PS1 mice received a first IN administration of MSC-CS and were tested 7 days later in the NORT (Fig. 4a), to verify if a single IN administration could replicate the memory recovery obtained with one IV injection. The treatment only partially rescued mouse memory (Fig. 4b), and was thus continued once weekly for one month. NORT 7 days after the end of treatment showed that memory was fully restored in APP/PS1 mice (Fig. 4c).

To verify if a regimen of regular administration is needed to guarantee the long-lasting functional effect, we re-tested these mice 39 days after treatment suspension. MSC-CS-treated mice showing a complete memory recovery one-week post-treatment were newly impaired (Fig. 4d) indicating that a sustained MSC-CS administration protocol is needed to preserve functional effects.

While we demonstrated that conditioning of MSC with AD brain homogenate is necessary to license the therapeutic features of the secretome, we cannot exclude that this effect might be related to inflammatory molecules present in the normal aged brain. Indeed, it is well-known that normal aging is associated with neuroinflammation [21, 22]. Accordingly, we have compared the licensing effect on MSC of brain homogenate from old WT mice (20 months of age) with that of young WT mice (6 months of age). Old APP/PS1 mice (22–25 months of age) were treated as above (4 weekly IN administrations) with the resulting MSC-CS (MSC-CS-WT old or MSC-CS-WT young). Upon testing in the NORT, there was an almost significant memory improvement in old APP/PS1 mice treated with MSC-CS-WT old (*P* = 0.063; Fig. 4e). In contrast, MSC-CS-WT young had no beneficial effect on memory loss in old APP/PS1 mice (Fig. 4f).

### Repeated IN MSC-CS treatment reduces neuropathology in 25-month-old APP/PS1 mice

As shown above, withdrawal of treatment led to re-establishment of memory impairment assessed at 39 days after treatment suspension in old APP/PS1 mice (Fig. 4d). Accordingly, and because the mice had tolerated the repeated MSC-CS administration well, we renewed the treatment with 4 additional IN administrations to re-potentiate MSC-CS effects, in order to evaluate how the treatment affected neuropathology.

**Fig. 5:**
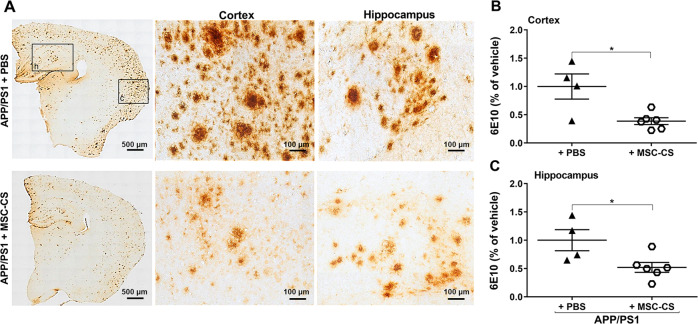
Repeated IN delivery of MSC-CS significantly decreases brain amyloidosis. **a** 6E10 immunostaining comparing brain plaque load between MSC-CS- and PBS-treated mice (left panels). In the middle and right panels, higher magnification of plaques in cortex (c) and hippocampus (h) showing MSC-CS-mediated changes in terms of aggregate density, and absence of the central plaque core. **b, c** Plaque quantification in the cortex and the hippocampus. Quantitative data are expressed as scatter plots with mean ± SEM. *P < 0.05, Student’s *t* test.

Mice were sacrificed 7 days after the 8th IN administration and their brains collected to assess multiple crucial targets. Figure 5a (left panels) shows plaque load reduction in the brain of aged MSC-CS-treated mice. The vast majority of residual plaques looked different in size and structure, lacking the typical dense nucleation core and with a lower density of aggregates (Fig. 5a middle and right panels). Quantitative analysis revealed a significant plaque reduction of 60% and almost 50% in the cortex (Fig. 5b) and hippocampus (Fig. 5c), respectively.

**Fig. 6:**
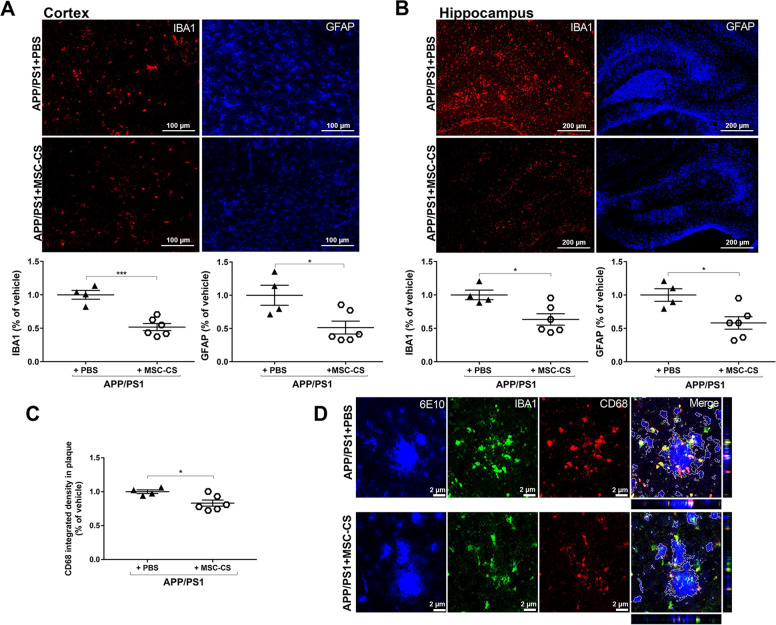
Repeated IN injections of MSC-CS significantly reduce neuroinflammation. **a**, **b** IBA1 and GFAP immunostaining showing the extent of gliosis in the cortex and the hippocampus between PBS- and MSC-CS-treated (8 IN) 25-month-old APP/PS1 mice (upper panels). Quantification of the IBA1- and GFAP-marked areas in the cortex and the hippocampus, respectively (lower panels). **c** CD68 quantification in plaques. **d** Immunofluorescence images of plaques (6E10), CD68 and IBA1 expression, and CD68-IBA1 co-localization (Merge) around plaques between treatment groups. Quantitative data are expressed as scatter plots with mean ± SEM. ***P < 0.001; *P < 0.05, Student’s *t* test.

Neuroinflammation was also significantly reduced by the treatment. Quantitative GFAP and IBA1 analysis demonstrated a close to 50% and 35% reduction in the cortex and hippocampus respectively (Fig. 6a, b). The phagocytic marker CD68 in the plaque area was also significantly lower in MSC-CS-treated mice (−26%; Fig. 6c). Of note, the decrease in the degree of microglia activation and CD68 expression also around plaques of similar size in MSC-CS-treated APP/PS1 mice was easily appreciable as compared to those treated with PBS (Fig. 6d), confirming the ability of MSC-CS administration to convert plaque structure/composition to a more inert state.

**Fig. 7:**
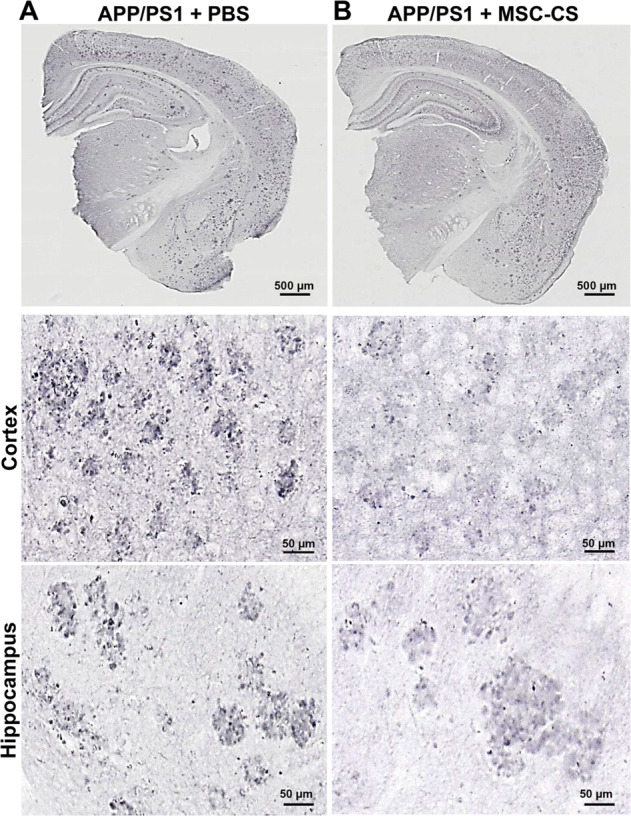
Repeated delivery of MSC-CS appears to reduce AβO load around plaques in APP/PS1 mice. Images of APP/PS1 mouse brain slices immunostained with the anti-AβOs A11 antibody showing the presence of AβOs in PBS- (**a**) and MSC-CS-treated (8 IN) 25-month-old mice (**b**); upper panels. Middle and lower panels depict a higher magnification of plaques in the cortex and the hippocampus of APP/PS1 mice showing AβO distribution (darker dots).

Because of the relevant neuropathogenic role played by Aβ oligomers (AβOs), the main neurotoxic species in AD [23] that is mostly determinant for glial activation and cognitive deficits [13, 24], we used immunostaining with the A11 anti-AβO antibody to assess if MSC-CS had modified their load. We found that APP/PS1 mice treated with MSC-CS appeared to have a much lower AβO load around plaques (Fig. 7a, b), which might explain the decrease in the phagocytic commitment of microglia and in the extent of neuroinflammation. Despite multiple attempts, we were not able to actually quantify the AβO specifically; indeed, although the oligomers show a darker, distinguishable staining, they overlap with the plaque halo, which could not be subtracted with Fiji software.

Double-staining A11-6E10 ascertained that the A11 antibody was not reacting with the entire plaque in an unspecific manner (Fig. S3A), but it marked only small aggregates surrounding plaques and some positivity was detectable also far from plaques (Fig. S3A). Notably, super-resolution microscopy revealed that AβOs are mainly engulfed in the lysosomes independently of the treatment (Fig. S3B).

### MSC-CS-treated 25-month-old APP/PS1 mice show increase neuronal density in both cortex and hippocampus and diminished hippocampal shrinkage

**Fig. 8:**
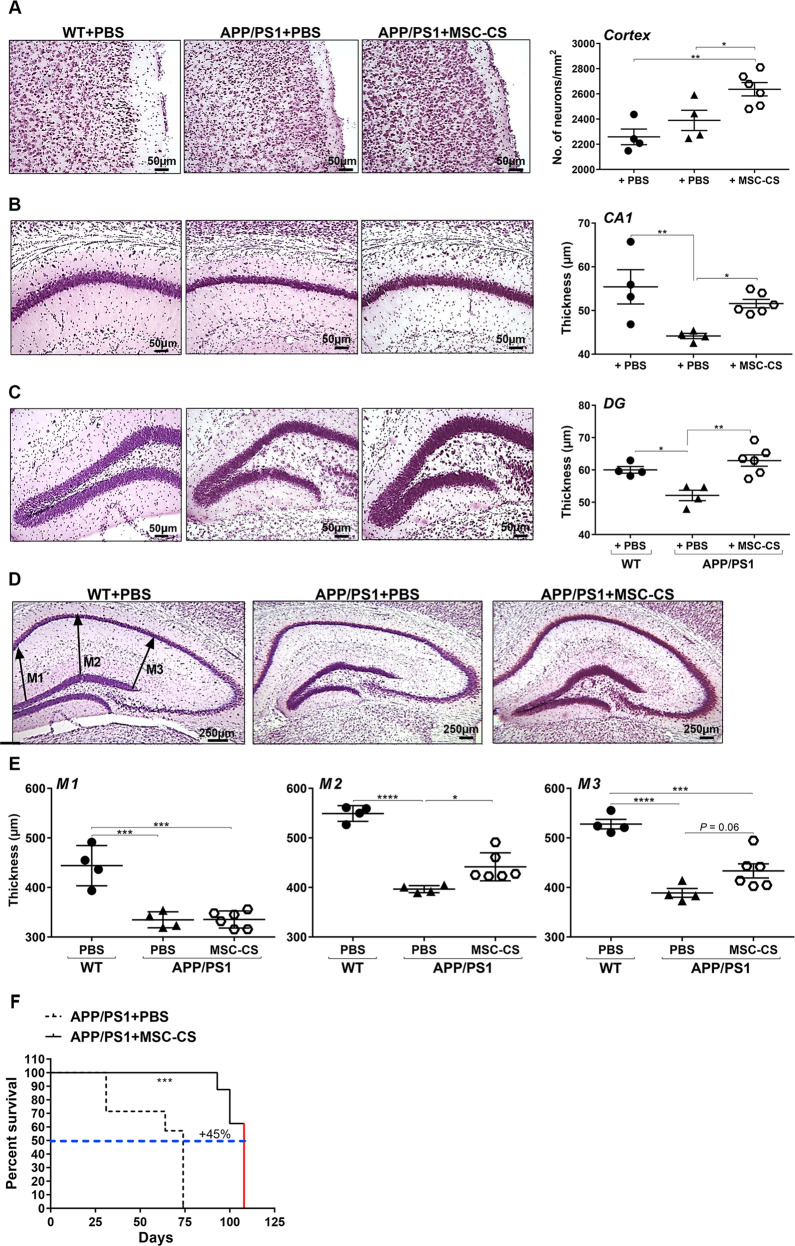
APP/PS1 mice treated repeatedly with MSC-CS display a higher number of neuronal cells in the cortex and the hippocampus, reduced hippocampal atrophy, and increased survival. Left panels are representative NISSL-stained sections of the cortex (**a**) and cell layer thickness in the CA1 region of the hippocampus (**b**) and the dentate gyrus (DG) (**c**). Right panels show neuronal quantification thereof. **d** Images of NISSL-stained brain slices in which headed arrows at M1, M2 and M3 indicate where thickness was measured between the CA1 and DG hippocampal sub-regions. **e** Quantification of neuropil thickness for the 3 zones selected. Data are expressed as scatter plots with mean ± SEM. One-way ANOVA; *P < 0.05, **P < 0.01; ***P < 0.001, ****P < 0.0001; Tukey’s multiple comparison post-hoc test. **f** The graph describes mouse longevity comparison between MSC-CS-treated and PBS-treated APP/PS1 mice. The red line in APP/PS1 + MSC-CS indicates that these mice were sacrificed for experimental needs, but were still perfectly healthy. ***P < 0.001, Student’s *t* test.

Because of the well-known neuroprotective action of MSC, we investigated if the treatment affected neuronal density in the cortex and hippocampus of our mice, by comparing NISSL-stained brain slices from PBS-treated WT vs. PBS- or MSC-CS-treated (8 IN) 25-month-old APP/PS1 mice. Through a well-established home-made macro [25], specifically counting neurons, we found that MSC-CS induced a significant 10% increase in neuronal number in the whole cortex (Fig. 8a). We observed an even higher, significant 25% increase in the perirhinal (PRH) and entorhinal (EC) cortex (Fig. S4), more critically affected in AD pathology [26] and involved in recognition memory [27], respectively.

In the hippocampus we measured the CA1 and dentate gyrus (DG) layer thickness. In PBS-treated APP/PS1 mice both layers were significantly thinner (−25.6% CA1; −15% DG) than in WT mice, whereas in MSC-CS-treated APP/PS1 mice, we appreciated a significant increment at both levels (+17% CA1;+21% DG) as compared to PBS-treated mice (Fig. 8b, c).

In addition, as read-out of hippocampal atrophy, we also systematically measured the neuropil thickness between the CA1 and DG subfields at 3 different levels (M1, M2, M3) in the same slice, and across the dorso-ventral hippocampal extension (brain anterior-posteriority from −1.90 to −2.70 from bregma). Hippocampal atrophy was particularly evident in PBS-treated APP/PS1 mice compared to WT (Fig. 8d), as confirmed by a significant reduction in M1 (−24.6%), M2 (−27.8%) and M3 (−26.3%) which was recovered by the treatment (Fig. 8e). We found that MSC-CS-treated mice had a significantly higher M2 neuropil thickness (+11.4%) and an almost significantly higher M3 (+11.4%) compared to that of WT mice. We did not detect any changes at the M1 level (Fig. 8d, e).

The neuroprotective effect of MSC-CS was corroborated by some upregulation, albeit not significant, of the mRNA expression of growth factors, such as brain-derived neurotrophic factor (BDNF), nerve growth factor (NGF) and growth-associated protein 43 (GAP43), in the brain from MSC-CS-treated APP/PS1 mice (Table 1). In addition, we found that mRNA expression of caspase 3, which reflects cellular apoptosis, was significantly reduced in MSC-CS-treated APP/PS1 mice as compared to PBS-treated mice (Student’s *t* test t_8_ = 2,3; *P* = 0.049; Table 1). These results suggest that MSC-CS is promoting mechanisms of neuronal protection, together with a reduced apoptosis in APP/PS1 mice, which could explain, at least in part, the increase in the number of neurons we observed.

**Table 1:** Comparison among APP/PS1 experimental groups for BDNF, NGF, GAP43 and Caspase 3 mRNA expression.

Gene	APP/PS1 + PBS	APP/PS1 + MSC-CS
Bdnf	−0.74 ± 0.09	−0.45 ± 0.27
Ngf	−0.64 ± 0.19	−0.43 ± 0.06
Gap43	−0.28 ± 0.42	−0.19 ± 0.06
Caspase 3	0.34 ± 0.03	0.20 ± 0.05*

*P < 0.05, Student’s *t* test

### APP/PS1 mouse lifespan is significantly increased by MSC-CS treatment

Repeated administrations (8 IN) of MSC-CS clearly prolonged the lifespan of 25-month-old APP/PS1 mice with a significant 45% increase compared to PBS-treated APP/PS1 mice (Fig. 8f). Two out of 7 PBS-treated APP/PS1 mice died 31 days after the first injection and another one after 64 days. The remaining 4 were sacrificed after 74 days after the first injection because their health conditions were no longer optimal, and their blood, CSF and brains were collected. In contrast, of the 9 mice receiving MSC-CS, 1 died 93 days after the first injection, 2 after 100 days and the last 6, still healthy, were sacrificed for neuropathology assessment 7 days after the end of the treatment, that is 105 days after the first injection.

## Discussion

We herein demonstrated that systemic injections of the MSC-CS collected from mouse bone marrow-derived MSC fully replicate cell effects described in AD mice [8, 9]. Sustained, MSC-CS intranasal administration in APP/PS1 mice fully restored mouse memory and impressively changed neuropathology at multiple crucial levels in very advanced disease stages. Although normal aging environment could foster the MSC-CS to assume an almost significant therapeutic effect, pre-conditioning of MSC to the specific AD microenvironment was fundamental to license them toward a significant neuroreparative, immunomodulatory phenotype. It was recently published that pre-conditioned MSC-derived exosomes restored memory and reduced inflammation when IV injected in APP/PS1 mice [28]. However, MSC were preconditioned through oxygen starving [28] rather than through a typical AD microenvironment as in our case. Our pre-conditioning more faithfully mimics the injured environment the cells would encounter, pointing to a specific AD-associated protective MSC activation.

Memory recovery is one of the major clinical outcomes to achieve with AD therapy. We strikingly demonstrated that, as observed with MSC themselves [8, 9], one IV injection of MSC-CS rescued memory after only 7 days from the injection, in 12- and 22-month-old APP/PS1 mice. Memory recovery was only transient, likely suggesting that MSC-CS bioactive components repairing the mechanisms implicated in synaptic function and memory processing rapidly vanish after a single treatment, hence the need for sustained treatment.

In 12-month-old mice, a single injection of MSC-CS induced a 30% plaque reduction and glial activation. In the brain of AD patients and AD mice, plaques are surrounded by activated glia [29] releasing pro-inflammatory cytokines responsible for synaptic/cognitive dysfunction [30, 31], and neurotoxicity [5]. In MSC-CS-treated mice, residual plaques were smaller in size and bordered by only a few microglial cells. Further assessment of the lysosomal glycoprotein CD68, a marker of active microglial phagocytosis [16, 32], confirmed a decrease in the phagocytic commitment of microglia in treated mice, indicating that remaining plaques were no longer chemoattractant. We cannot yet establish which plaque clearance mechanism/s were stimulated by MSC-CS. Previous studies demonstrated that MSC co-cultured with Aβ-treated neurons increased the number of LC3-II-positive autophagosomes co-localizing with lysosomal markers, an aspect which could be replicated by MSC-CS explaining plaque decrease [33]. However, Aβ clearance relies on the activation of several systems [34], thus dedicated studies are needed to clarify this issue.

We also demonstrated that MSC-CS partially reduced the pro-inflammatory astrocytic phenotype, by significantly decreasing their TNFα expression. New data demonstrated that the two glial cells are detrimentally interconnected with activated microglia promoting synapse loss, cognitive deficits [35], and an A1 astrocytic neurotoxic phenotype, with TNFα as a crucial player [19]. Our findings demonstrate an association of MSC-CS-mediated positive effects leading to a reduction in plaque load and microglia activation, which resulted in restored memory and reduced A1 phenotype. However, this might be only partially true since one IV MSC-CS injection in aged mice did not modify brain neuropathology, despite an impressive memory recovery. Although we have no explanation for this, we assume that when the pathology is more severe, one MSC-CS injection is insufficient to modify neuropathology. In contrast, it appears that independently of plaque or glial changes, some secretome component/s directly act at neuronal level allowing regular memory processing. As aforementioned, this hypothesis is supported also by the fact that in the younger treated mice, memory recovery observed 7 days post-injection vanished 5 days later despite plaque reduction. These data are in agreement with the notion that plaque load is no longer considered as a correlate of synaptic and cognitive dysfunction, which has been functionally attributed to the soluble oligomeric Aβ species [36].

Failure of a single IV MSC-CS injection to exert a beneficial effect at neuropathological level in older mice was overcome with a sustained IN treatment. IN administration is an alternative route [37], currently widely used for MSC or exosome administration with favorable outcomes [38–40].

We demonstrated in the NORT that MSC-CS induced full memory recovery after four IN injections; this functional effect was also subsequently confirmed under different treatment regimens in the Y-maze alternation test (data not shown). In addition, regular weekly MSC-CS administrations significantly reverted neuropathology in 25-month-old APP/PS1 mice.

As an added value, we also proved that MSC conditioning strongly influences the therapeutic efficacy of the secretome. Indeed, lack of cell pre-conditioning failed to promote the therapeutic activity of the secretome. Moreover, the pre-conditioning effect could be age-related, reaching its maximal efficacy in presence of AD pathology. Indeed, MSC exposed to young WT mouse brain homogenate yielded MSC-CS with no reparative effect in 25-month-old APP/PS1 mice, whereas MSC-CS-WT old exerted an almost significant reparative effect on the impaired memory of 22-month-old APP/PS1 mice. It is well known that normal aging is associated with neuroinflammation [21, 22]; our findings therefore suggest that inflammatory molecules, which are absent, or present in minimal amounts, in young brain are indeed relevant for the licensing of the neuroprotective effect of MSC that reaches its maximum expression when conditioning is induced with AD-related stimuli. A large number of studies clearly indicate that the environment in which MSC operate licenses their plasticity [41], and implicates diverse molecules. For example, an inflammatory environment mimicked by exposure to inflammatory cytokines is essential to promote the immunosuppressive function of MSC on T cells [42]. Although we cannot herein postulate what AD-related factor(s) are involved in priming our MSC, we assume that multiple pathology-related components such as the presence of Aβ aggregates, pro-inflammatory cytokines and chemokines could be implicated.

A healthier state in the 25-month-old APP/PS1 mice receiving sustained MSC-CS treatment with respect to age-matched APP/PS1 mice receiving PBS was observed. The latter started to die about two months before the MSC-CS group and none of them survived until the end of the experiment. In contrast, 70% of MSC-CS mice were still alive showing a wellbeing state and were sacrificed for neuropathological assessment. Increased survival was also described in a murine sepsis model treated with MSC preconditioned with staphylococcal enterotoxin B [43]. A clinical and neuropathological amelioration was observed in mice modeling multiple sclerosis treated either with MSC themselves [44, 45], or with their secretome [46]. In amyotrophic lateral sclerosis-affected mice, administration of MSC intravenously improved survival and motor function [44, 46, 47].

IN-repeated MSC-CS administration in APP/PS1 mice from the age of 22 up to 25 months also induced a dramatic reduction in cortical and hippocampal plaque load, in the number of surrounding activated glial cells and in the expression of the phagocytic marker CD68. A further explanation for this latter outcome was the lower concentration of AβOs in residual plaques. Plaques act as reservoirs of AβOs, the smaller, soluble Aβ species which freely circulate perturbing neuronal and glial activity [4, 23, 48]. Notably, in an AβO-induced acute mouse model [49], we showed that AβOs specifically induce a reversible memory impairment through glial activation, and anti-inflammatory drugs prevent it [13, 50]. In our APP/PS1 mice, AβOs were all mainly distributed within the plaque corona. Remarkably, super-resolution analysis found them all engulfed in CD68-positive lysosomes in both PBS- and MSC-CS-treated mice. Thus, we assume that microglial cells permanently engulf AβOs in the attempt to limit plaque growth or remove them from the brain; upon MSC-CS treatment, the lower AβO concentration results in decreased microglia phagocytic commitment and reduced neuroinflammation. The ability of MSC-CS to lower the level of AβOs is an incommensurable therapeutic potential in a translational prospective since AβOs are the foremost target to counteract [23, 48].

Of note, MSC-CS also reduced hippocampal atrophy and neuronal damage in the brain of APP/PS1 mice. Brain atrophy is another core feature of AD [51], but to date no data were shown in this regard on the effects of MSC in mice. Patient brains are typified by gross neuronal loss. The entorhinal cortex is the first affected area followed by the subiculum and the CA1 sub-region of the hippocampus up to cortical areas [52]. We detected a significant hippocampal shrinkage and thinner pyramidal layers in both the CA1 and DG in PBS-treated APP/PS1 mice compared to WT, no longer detectable in APP/PS1 receiving MSC-CS. Accordingly, the neuropil thickness between the CA1 and DG pyramidal cell layer was significantly larger than in APP/PS1 mice receiving PBS. Both CA1 and DG neuronal cell layers showed a significantly higher cell density compared to PBS-treated mice. A much higher number of cells in the cortex of MSC-CS-treated compared to WT and PBS-treated APP/PS1 mice was also highlighted, accompanied by a decrease in mRNA expression of the apoptosis marker Caspase 3, and a trend in increased mRNA expression of nerve growth factors. These outcomes might depend on the ability of MSC-CS to protect neurons preventing their loss. Previous in vitro studies demonstrated that MSC-derived secretome promotes neuronal survival and increases metabolic activity of a stably transfected human cell model of AD [53]. An increase in neurogenesis mediated by MSC was also described in Aβ-induced cells and AD mice [54]. It is also conceivable to assume that glial cell activation reduction might have prevented neuronal death. New investigations are ongoing to clarify these aspects in our mice.

In conclusion, we proved for the first time that the secretome derived from AD-conditioned MSC can fully replicate the neuroreparative actions previously described with MSC direct applications. The MSC-CS effects were impressive under longer treatments. Notably, besides plaque reduction the treatment also reduced their AβO concentration, and despite the absence of cell implantation it abolished brain atrophy in crucial affected areas.

The paracrine action of MSC is becoming a certainty. The possibility to replace the use of stem cells with the safer application of those bioactive components cells would release in the host, represents an invaluable opportunity for the therapy of neurodegenerative diseases. Our study clearly proves the great therapeutic potential of MSC-CS and poses the essential bases for the future identification of the multiple mechanisms that underlie the therapeutic mode of action of MSC-CS and of the secretome components needed to repair AD brains. The tangible possibility, indeed, to subsequently manufacture these components will permit to prepare to-go biologic products opening a new horizon in the clinical use of MSC.

## Materials and methods

### Mice

APPswe/PS1Δe9 (APP/PS1) transgenic male mice [B6C3- Tg(APPswe,PSEN1dE9)85Dbo/Mmjax mice] and age-matched wild type (WT) littermates were purchased from Jackson Laboratories (USA). No environmental enrichment was used since it notably improves AD pathology in mouse models of AD [55, 56]. Mice were all drug and behavioral test naïve and the experiments were all conducted during the light cycle. All animals were housed in a SPF facility in groups of 4 in standard mouse cages containing sawdust with food (2018S Envigo diet) and water ad libitum, under conventional laboratory conditions (room temperature: 20 ± 2 °C; humidity: 60%) and a 12/12 h light/dark cycle (7:00 am – 7:00 pm).

The IRFMN adheres to the principles set out in the following laws, regulation, and policies governing the Care and Use of Laboratory Animals: Italian Governing Law (D.lgs 26/2014; Authorization n.19/2008-A issued March 6, 2008 by Ministry of Health); Mario Negri Institutional Regulations and Policies providing internal authorization for persons conducting animal experiments (Quality Management System Certificate – UNI EN ISO 9001:2015 – Reg. N° 6121); the NIH Guide for the Care and Use of Laboratory Animals (2011 edition) and EU directives and guidelines (EEC Council Directive 2010/63/UE). The statement of Compliance (Assurance) with the Public Health Service (PHS) Policy on Human Care and Use of Laboratory Animals has been reviewed (9/9/2014; Animal Welfare Assurance #A5023-01).

### APP/PS1 brain homogenate preparation

The brains of young or old WT, or those of APP/PS1 mice aged- and sex-matched to those undergoing the treatment were collected and homogenized on ice in DMEM (100 mg/mL; GIBCO). The homogenate was thus centrifuged at 12.000 rcf for 20 min at 4 °C and the supernatant collected and frozen in liquid nitrogen and stored at −80 °C until use.

### Preparation of MSC and secretome thereof

Murine bone marrow-derived MSC were obtained and characterized as described previously [44], and cultured (2 × 10^6^ cells in 10 mL medium in T75 flask) in supplemented Mesencult medium (StemCell Technologies) following manufacturer’s instructions. MSC were negative for mycoplasma contamination. MSC were then incubated first in “starvation medium” (serum-free RPMI containing glutamine) for 30 min, followed by stimulation with 10% APP/PS1 mouse brain homogenate in Mesencult medium for 24 h. Upon removal of the stimulation medium, MSC were incubated in starvation medium for 24 h, upon which the culture supernatant (MSC-CS) was collected and concentrated using Amicon Ultra centrifugal filters (Amicon Ultra-15, Millipore) to a volume equivalent to the secretome of 1 × 10^6^ MSC in 200 µl for intravenous (IV) injection. Since the maximum intranasal (IN) administration volume is 25 µL, MSC-CS was concentrated by ultrafiltration to maintain the same IV MSC-CS dose (equivalent to 1 × 10^6^ MSC/200 µL) in 25 µL.

### MSC treatment and memory evaluation

MSC were infused IV at 1 × 10^6^ dose in a volume of 200 µL. Mice were tested in the NORT 30 days post-injection.

### Experimental groups for MSC-CS treatment

#### Group 1

Twelve-month-old APP/PS1 mice received one IV injection of either PBS (*n* = 6), MSC-CS (*n* = 5) or MSC-UCS (*n* = 6). Seven days later were tested in the NORT. Age-matched WT received one IV injection of PBS (*n* = 7).

#### Group 2

Twelve-month-old APP/PS1 mice and age-matched WT received one IV injection of PBS or MSC-CS (WT + PBS *n* = 14; WT + MSC-CS *n* = 6; APP/PS1 + PBS *n* = 13; APP/PS1 + MSC-CS *n* = 13). Seven days later mice were tested in the NORT. Seven of them were then sacrificed for brain collection and neuropathological analysis the day after the test, the other were re-tested 14 days later to verify effect duration. This experiment was replicated twice.

#### Group 3

Twenty-two-month-old APP/PS1 mice received one IV injection of MSC-CS. Seven days post-injection they were tested in the NORT and the day after killed for brain collection and neuropathology assessment at a more advanced stage of disease compared to group 1 and 2.

#### Group 4

Twenty-two month-old APP/PS1 and age-matched WT underwent a repeated intranasal (IN) treatment regimen (25 µL/inj, 1 inhalation/week) and were tested in the NORT 7 days after the first and fourth inhalation and again 30 days after the fourth inhalation (WT + PBS *n* = 6; APP/PS1 + PBS *n* = 7; APP/PS1 + MSC-CS *n* = 9). Mice were then re-treated with 4 further weekly inhalations. Neuropathology was assessed 7 days after the eighth inhalation.

#### Group 5 and 6

Twenty-two-month-old and 25-month-old APP/PS1 and age-matched WT mice underwent a repeated IN treatment regimen (25 µL/inj, 1 inhalation/week) with MSC-CS collected from MSC exposed to brain homogenate derived from 20-month-old or 6-month-old WT mice, respectively. Mice were tested in the NORT 7 days after the fourth inhalation (22-month-old: WT + PBS *n* = 6; APP/PS1 + PBS *n* = 6; APP/PS1 + MSC-CS *n* = 6; 25-month-old: WT + PBS *n* = 6; APP/PS1 + PBS *n* = 7; APP/PS1 + MSC-CS *n* = 6).

### NORT

Mice were tested in an open-square gray arena (40 × 40 cm), 30 cm high as previously described [57]. The task started with a 5-min habituation trial. The next day, mice were again placed in the arena containing two identical objects (familiarization phase). Exploration was recorded in a 10-min trial. Twenty-four hours later (test phase) mice were again placed in the arena containing two objects: one familiar and a new one, and the time spent exploring the two objects was recorded for 10 min. Memory was expressed as a discrimination index (DI), i.e. (seconds on novel – seconds on familiar)/(total time on objects). Mice exposed to NORT for the second time were not subjected to the habituation trial.

#### Immunohistochemistry for amyloid plaques, AβOs or CD68

Mice were anesthetized with a mixture of medetomidine/ketamine and intracardially perfused with phosphate buffer saline (PBS); their brains were removed, post-fixed in 4% paraformaldehyde and stored at −80 °C after cryo-protection. APP/PS1 mouse brain coronal sections (20 μm) were cut using a Leica cryostat and incubated for 1 h at room temperature (RT) with 10% normal goat serum (NGS) then overnight (O/N) at 4 °C with primary antibodies (6E10 1:500; Signet, Cat. # 803001; A11 1:1000; Invitrogen, Cat. # AHB0052; CD68 1:200; Biorad, Cat. # MCA-1957). After incubation with the appropriate biotinylated secondary antibodies (1:200; Vector Laboratories), immunostaining was developed using the avidin-biotin kit (Vector Laboratories) and diaminobenzidine (Sigma, Italy) as chromogen. Only for the A11 staining DAB was amplified with nickel.

#### Immunofluorescence

Based on the analysis needed, 20 μm APP/PS1 cryostat coronal brain sections were collected in 100 mM PBS and co-immunostained with a maximum of 3 primary antibodies concomitantly. Plaques, CD68 and A11 were stained as described above. Microglial cells with anti-Iba1 (Iba1: 1:1000, O/N; Wako, Cat. # 019–19741) and astrocytes with anti-GFAP (1:3500, O/N; Chemicon Int. Inc., Cat. # MAB3402). We immunostained also IL-1β (1:200, Santa Cruz Biotechnology, Cat. # SC-1252; 72 h, 4 °C) and TNFα (1:100; Tebu-Bio, Cat. # 500-P644-50UG; 72 h, 4 °C) co-localized with astrocytes and microglia. These protocols were all previously described [32, 50]. The primary antibodies were followed by the application of the appropriate fluorescent secondary antibodies (1:500 for 1 h at RT) conjugated respectively with Alexa 488, 546 or 647 (Molecular Probes). In the case of IL-1β and TNF-α a streptavidin Tyramide Signal Amplification kit (NEN Life Science Products) was used with the secondary antibody. Immunofluorescence was acquired using an IX81 microscope and a FV500 confocal scan unit with three laser lines [argon-krypton (488 nm), helium-neon red (646 nm), and helium-neon green (532 nm; Olympus)] and an ultraviolet diode.

#### NISSL staining

Twenty-µm slices were collected with a Leica cryostat on gelatin-coated slides for a total thickness of 760 µm - from bregma −1.94 to −2.70 - selecting one slice every other 5. After 2-day drying and a 1-min wash in H_2_O, slices were dehydrated with EtOH (70%, 95%, 100%, 5-min each), until xylene (5-min). Slices were processed backward until cresyl violet solution (3-min), followed by H_2_O + 70% EtOH washes and 3-min in EtOH/acetic acid. Following 100% EtOH and xylene (5-min/each), the slices were covered with a glass microcover (Prestige) using DPX mountant.

### Image analysis

Quantitative analyses were done by an operator blind to genotype and treatment and normalizing on the quantified area. Area of selection for image analysis were the whole hippocampus and/or the whole cortex of a single hemisphere. We analyzed 3 20-µm thick slices/mouse. Brain images were acquired using the Olympus Virtual Stage microscope. Immunofluorescence images were acquired using the Olympus confocal microscope; the acquisition setting was standardized for each analyzed marker and applied throughout the study. The brain area of interest was selected in bright field and the immunoreactivity for each specific marker was quantified by applying dedicated home-made macros through Fiji software [32, 50, 58].

### Gene expression analysis

Total RNA was extracted from hippocampi by PureLink RNA Mini Kit (Invitrogen) following the manufacturer’s instructions. Samples were treated with DNase I (Invitrogen) and reverse-transcribed with random hexamer primers using cDNA Reverse Transcription Kit (Applied Biosystem). The analysis was performed using Power syber green PCR master mix (Applied Biosystem) and 7300 Real-Time PCR System (Applied Biosystems). mRNA expression of target genes was evaluated using primers listed in Table 2. RPL27 was used as housekeeping gene to normalize mRNA levels. Relative gene expression was determined by ΔΔCt method. Data are expressed as log2 of the fold difference from the control group.

**Table 2:** List of primers used for real-time reverse transcription polymerase chain reaction.

Gene	Forward Primer	Reverse Primer
Bdnf	AGGCACTGGAACTCGCAATG	AAGGGCCCGAACATACGATT
Ngf	CCAAGGACGCAGCTTTCTAT	CCTGTACGCCGATCAAAAAC
Gap43	CAGAGGATGCTGCCACCAA	GTTTGGCTTCGTCTACAGCG
Casp3	GGTTCATCCAGTCCCTTTGC	CTAGCTTGTGCGCGTACAGC

### Structured illumination microscopy (SIM)

SIM analysis of brain sections was performed on a Nikon SIM system. Tissues were imaged at laser excitation of 488 (for A11), 561 (for CD68) nm with a 3D-SIM acquisition protocol. Sixteen-bit images sized 1024×1024 pixels with a single pixel of 0.030 µm were acquired in a gray-level range of 0–16000 to exploit the linear range of the camera (iXon ultra DU-897U, Andor) and to avoid saturation. Three-dimensional Z-stacks were scanned with a 0.125 µm step size over 2–3 µm. Raw and reconstructed images were validated with the SIM-check plugin of ImageJ [59] and only those providing satisfactory image diagnosis were included in the study. Images were finally elaborated with GIMP (Gnu Image Manipulation Program).

### Statistical analysis

Data were expressed as scatter plots with mean ± standard error of the mean (SEM) and analyzed using GraphPad Prism software. All data reported in the paper met the assumption of normal distribution. The estimated variance for NORT data was of 0.15, whereas for histological biomarker analysis it was around 0.1. Notably, the variance we obtained was very similar between statistically compared experimental groups. We used either one-way or two-way analysis of variance (ANOVA) based on the experimental design. In the presence of a significant effect of treatment (one-way ANOVA), or a significant interaction between transgene and treatment (two-way ANOVA), appropriate post-hoc tests were applied. The Student *t* test was used for a comparison between two experimental groups. A *P* value < 0.05 was considered significant. Sample size of 6–8 mice/group was estimated on the basis of our wide experience with APP/PS1 mice. Experimental mouse groups with a lower number of mice (i.e. 4 or 5) are due to mouse exclusion or mouse death throughout the study. Exclusion criteria were pre-established as follows: (i) exclusion of mice from the NORT was applied only in the case of mouse immobility during either one of the test phases; (ii) the total time of object exploration was lower than 10 s. Exclusion of samples for histological analysis was applied only in the case of brain tissue damage and inability to obtain quantifiable immuno-labeling. Mice were randomly selected from each cage to assign half of the animals to the control group and the other half to the treatment group. All the in-vivo and ex-vivo analyses were conducted in blind.

## Supplementary information

Suppl. Figure legends

Suppl. Fig. 1

Suppl. Fig. 2

Suppl. Fig. 3

Suppl. Fig. 4
