# Supplementary material for: Intranasal delivery of mesenchymal stem cell secretome repairs the brain of Alzheimer’s mice
Source: Cell Death Differ. 2020 Jul 23;28(1):203–18. doi: 10.1038/s41418-020-0592-2 (PMC7852675; doi:10.1038/s41418-020-0592-2)
Supplement: Supplementary file 1 — Suppl. Figure legends [file 41418_2020_592_MOESM1_ESM.docx]

Supplementary figure legendS

**Fig. S1. One MSC IV injection restores mouse memory in APP/PS1 mice.** Discrimination index of 11-month-old APP/PS1 mice tested in the NORT one month post-MSC (1x10^6^/200 uL) IV injection. Data are expressed as scatter plots with mean ± SEM. One-way ANOVA; *P<0.05, Tukey’s post-hoc test.

**Fig. S2. One MSC-CS IV injection restores memory in old APP/PS1 without affecting neuropathology.** (**A**) Experimental design. (**B**) Discrimination index of 22-month-old APP/PS1 mice tested in the NORT 7 days post-MSC-CS injection. One-way ANOVA; ***P<0.001, ****P<0.0001 Tukey’s post-hoc test. **(C)** 6E10 immunostaining comparing brain plaque load between treated and untreated mice (upper panels). Plaque quantification in the cortex and the hippocampus (lower panels). Quantitative data are expressed as scatter plots with mean ± SEM. *P<0.05, Student’s t-test. **(D)** IBA1 (microglia), GFAP (astrocytes) and TNFα expression in the hippocampus of either PBS- or MSC-CS treated APP/PS1 mice.

**Fig. S3. The A11 antibody well distinguishes AβOs from plaques.** (A) Co-localization between the anti-AβOs A11 antibody (green) and the 6E10 antibody marking amyloid plaques (blue). (B) Super-resolution (SIM) and confocal microscopy images showing AβO (A11, red) engulfment in lysosomes (CD68, green).

**Fig. S4. MSC-CS repeatedly-treated APP/PS1 display increased neuronal density in entorhinal and perirhinal cortex.** Cumulative neuronal quantification of NISSL-stained entorhinal and perirhinal cortex. Quantitative data are expressed as scatter plots with mean ± SEM. **P<0.01; *P<0.05, Tukey’s post-hoc test.
