# Supplementary figures and images for: Intranasal delivery of mesenchymal stem cell secretome repairs the brain of Alzheimer’s mice
Source: Cell Death Differ. 2020 Jul 23;28(1):203–18. doi: 10.1038/s41418-020-0592-2 (PMC7852675; doi:10.1038/s41418-020-0592-2)

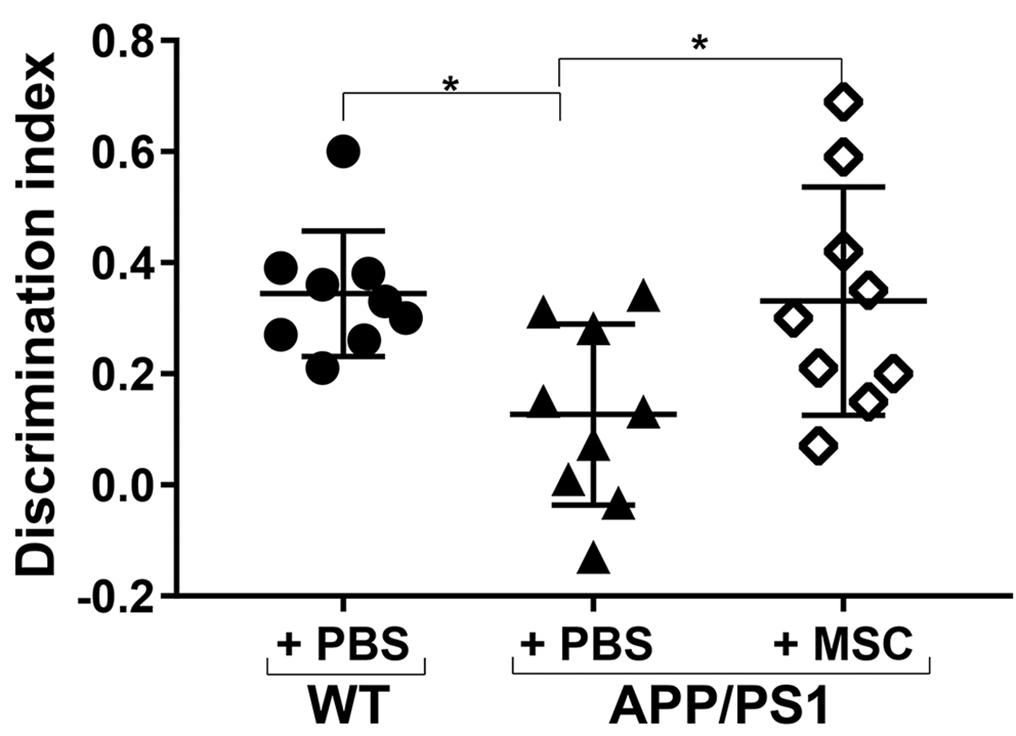

Supplement: Supplementary file 2 — Suppl. Fig. 1 [file 41418_2020_592_MOESM2_ESM.tif]

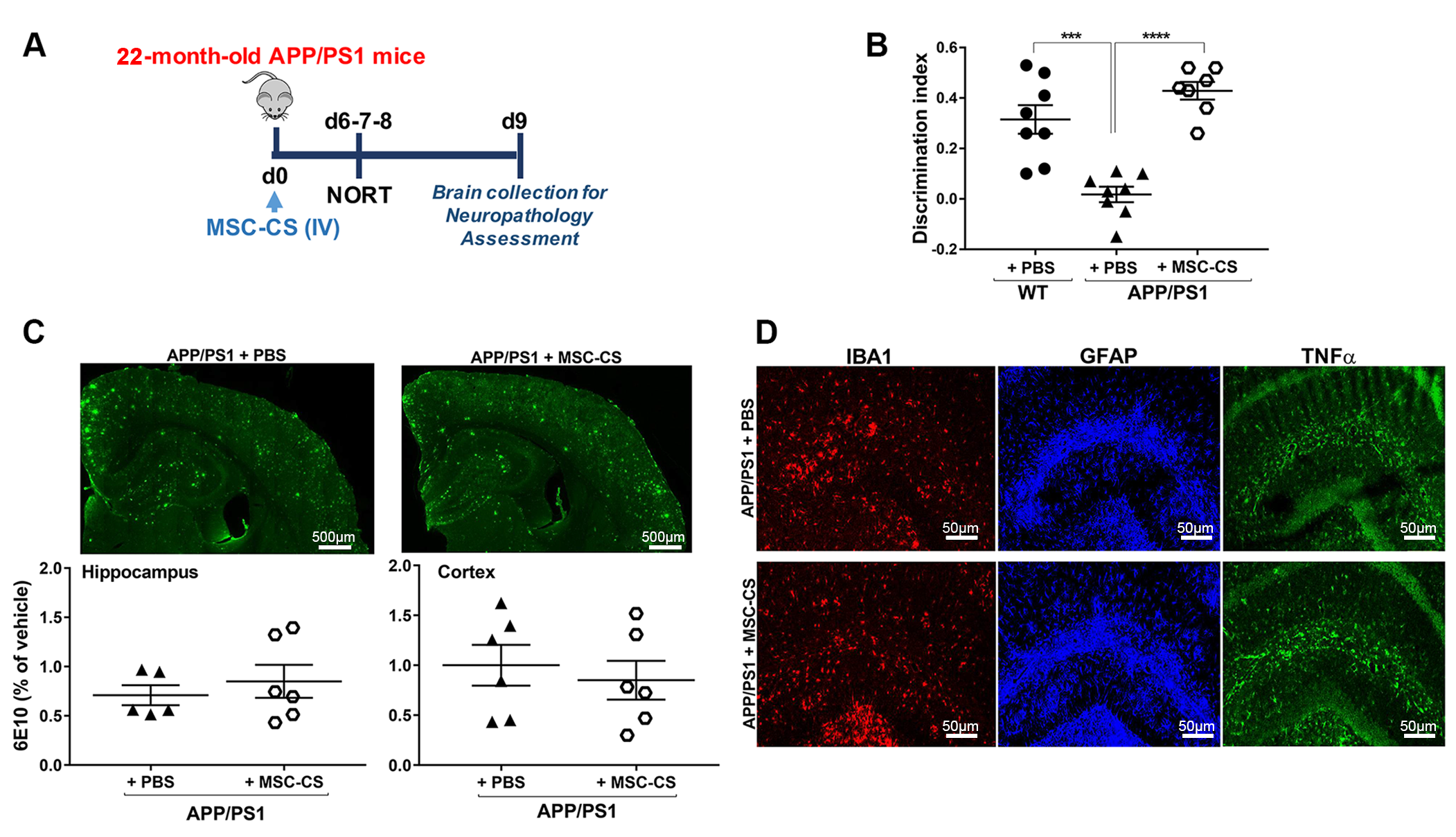

Supplement: Supplementary file 3 — Suppl. Fig. 2 [file 41418_2020_592_MOESM3_ESM.tif]

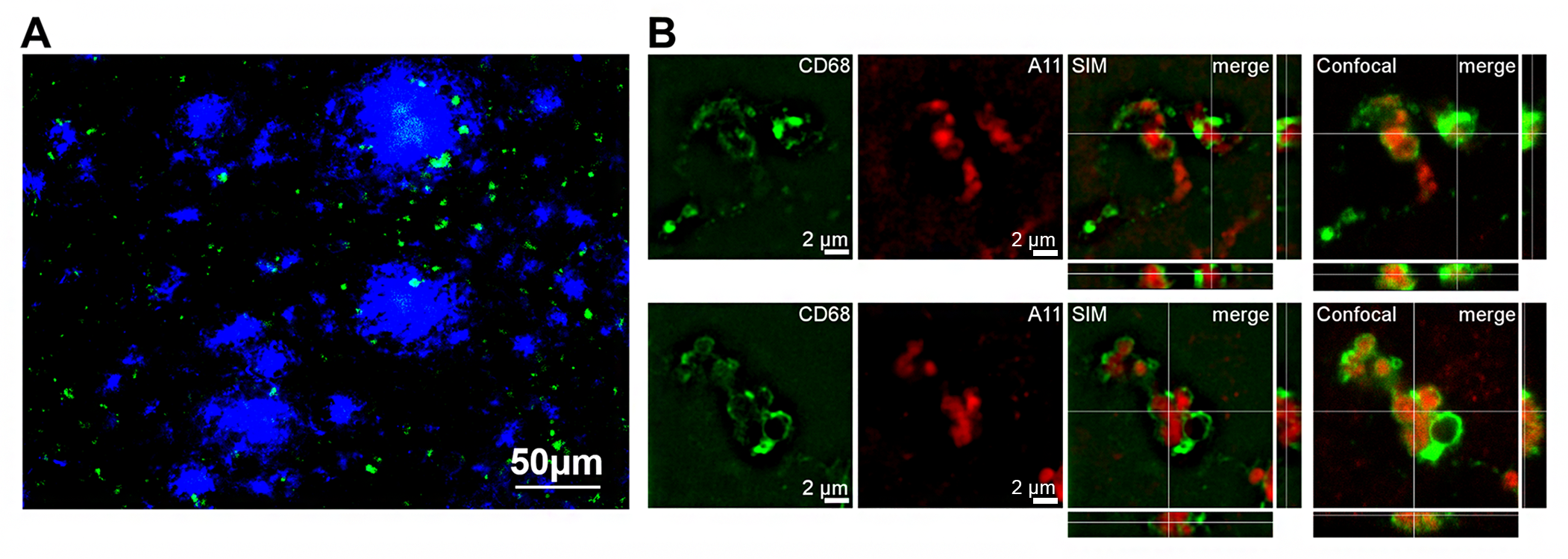

Supplement: Supplementary file 4 — Suppl. Fig. 3 [file 41418_2020_592_MOESM4_ESM.tif]

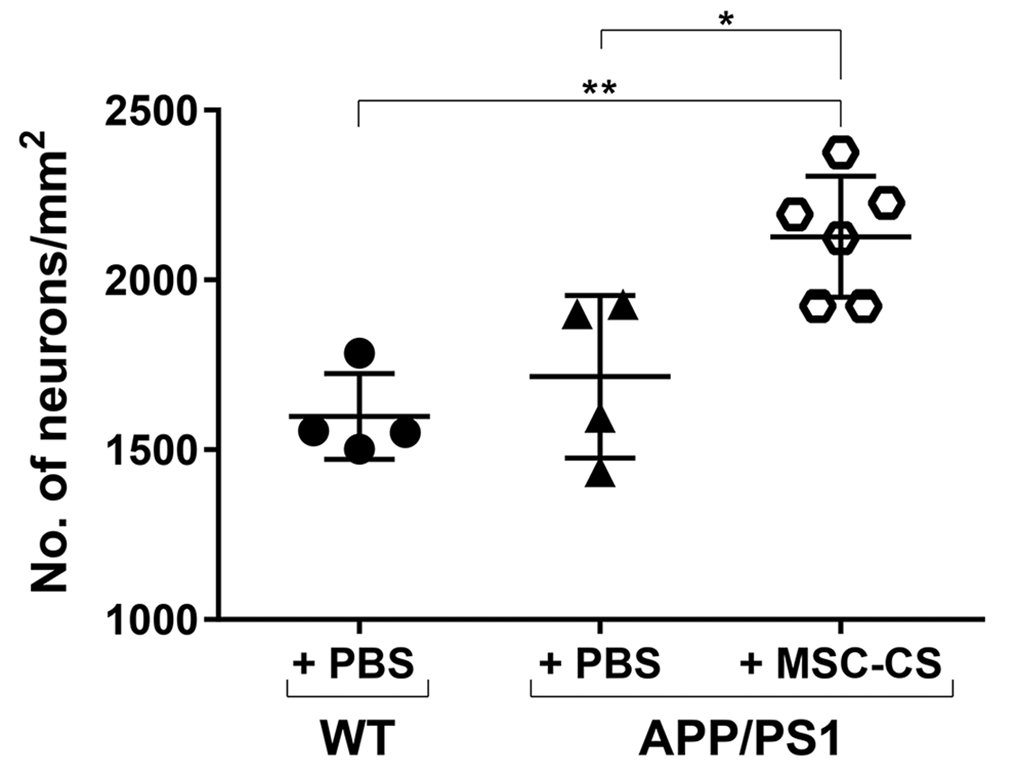

Supplement: Supplementary file 5 — Suppl. Fig. 4 [file 41418_2020_592_MOESM5_ESM.tif]
